# Introduction to the special issue Amphibian immunity: stress, disease and ecoimmunology

**DOI:** 10.1098/rstb.2022.0117

**Published:** 2023-07-31

**Authors:** Vania Regina Assis, Jacques Robert, Stefanny Christie Monteiro Titon

**Affiliations:** ^1^ Departamento de Fisiologia, Instituto de Biociências, Universidade de São Paulo, 05508-900 São Paulo, Brazil; ^2^ College of Public Health, University of South Florida, Tampa, FL 33612-9415, USA; ^3^ Department of Microbiology and Immunology, University of Rochester, Rochester, NY 14642, USA

**Keywords:** chytridiomycosis, corticosterone, infection, microbiome, pathogen, ontogeny

## Abstract

Amphibian populations have been declining worldwide, with global climate changes and infectious diseases being among the primary causes of this scenario. Infectious diseases are among the primary drivers of amphibian declines, including ranavirosis and chytridiomycosis, which have gained more attention lately. While some amphibian populations are led to extinction, others are disease-resistant. Although the host's immune system plays a major role in disease resistance, little is known about the immune mechanisms underlying amphibian disease resistance and host–pathogen interactions. As ectotherms, amphibians are directly subjected to changes in temperature and rainfall, which modulate stress-related physiology, including immunity and pathogen physiology associated with diseases. In this sense, the contexts of stress, disease and ecoimmunology are essential for a better understanding of amphibian immunity. This issue brings details about the ontogeny of the amphibian immune system, including crucial aspects of innate and adaptive immunity and how ontogeny can influence amphibian disease resistance. In addition, the papers in the issue demonstrate an integrated view of the amphibian immune system associated with the influence of stress on immune–endocrine interactions. The collective body of research presented herein can provide valuable insights into the mechanisms underlying disease outcomes in natural populations, particularly in the context of changing environmental conditions. These findings may ultimately enhance our ability to forecast effective conservation strategies for amphibian populations.

This article is part of the theme issue ‘Amphibian immunity: stress, disease and ecoimmunology’.

## 1. Introduction

Amphibian population declines and extinctions have been reported worldwide [1,2]. In addition to environmental changes like temperature shifts, changes in rainfall regimes, loss of habitats, fragmentation, pollution, infectious diseases significantly contribute to this scenario [2–7]. In addition, there are a multitude of documented shifts in behaviour and physiology that occur in native populations dealing with climate change and infectious diseases, which have been associated with alterations in hormonal and immune modulation, possibly leading to systemic failure and death [8–13]. Among physiological changes, alterations in stress-related measures such as increased glucocorticoid levels may have critical implications on disease resistance and host survival [7,9,11,13,14].

One physiological aspect that has been recently observed in some outbreaks is the increase in amphibian plasma glucocorticoid levels, signalling a stress response. In the last decades, studies have been showing that habitat alterations and fragmentation, changes in temperature, and dehydration are associated with increased corticosterone levels, the main glucocorticoid in amphibians [8–10,15–17]. In addition, increased pathogenicity of the fungus *Batrachochytrium dendrobatidis* (*Bd*) and ranavirus has been positively correlated with corticosterone levels in some amphibian species [11,18–22], pointing to a possible glucocorticoid influence on the stress-related augmented mortality in diseased individuals.

Stress-induced immunomodulation in vertebrates has been widely investigated in the last decades, with acute stress response being associated with immunoenhancing and chronic stress with immunosuppressive effects [23–26], including in amphibians [27–33]. In addition, the general effects of stressors and their primary mediator (corticosterone) on amphibian immunity have been suggested as a potential driver of amphibian resistance/vulnerability to pathogens and habitat alterations [7,11,18]. Therefore, understanding the health-related implications of stress-induced immunomodulation and its endocrine associations in amphibians under acute/short-term and chronic/long-term conditions will bring a better idea of the consequences of environmental stressors on amphibian immunity. Moreover, determining how and which physiological mediators are involved in natural and anthropogenic-induced stress responses is of great importance for the physiology, ecology, ecoimmunology and conservation biology of amphibians.

**Figure 1.**
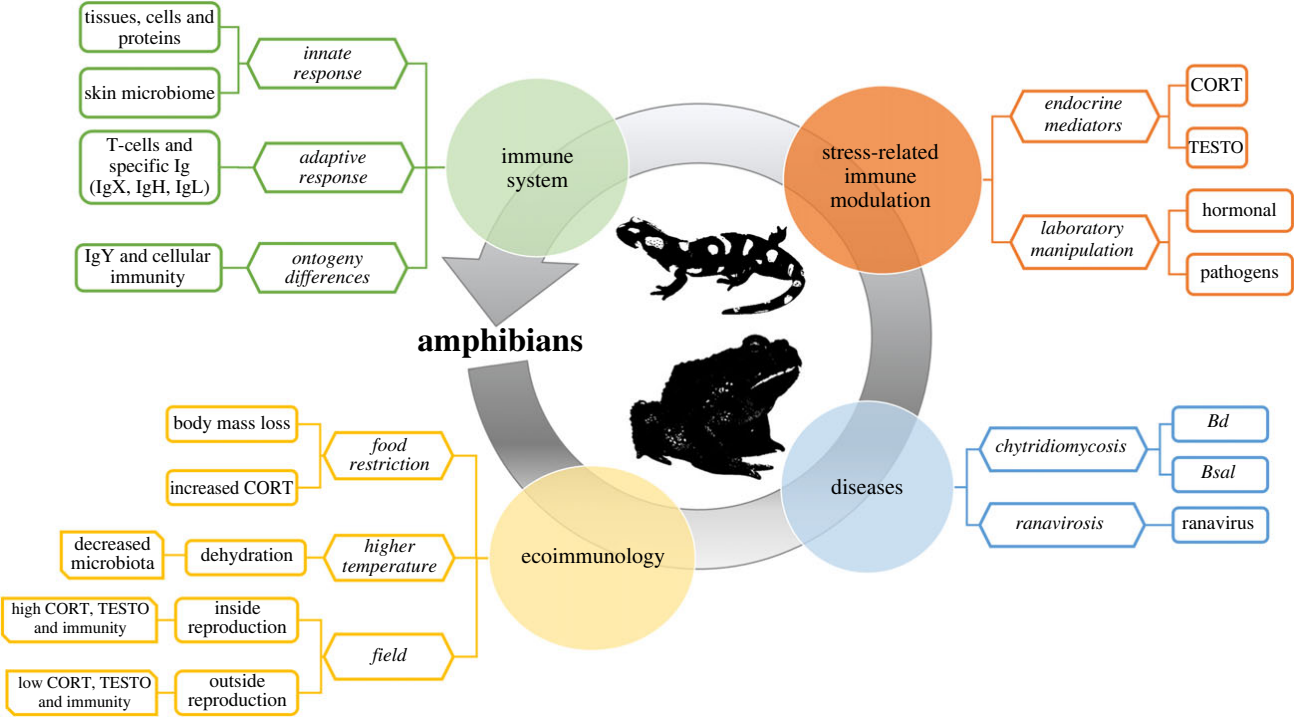
A representative figure of how the papers in the present issue contribute to the distinct approaches to investigating amphibian immunity. Ig: antibodies; CORT: corticosterone; TESTO: testosterone. (Online version in colour.)

The papers in this theme issue focus on what has recently been learned about amphibian immunity and its endocrine and environmental modulation (figure 1). Each study helps us to better define how environmental changes, stress physiology and disease resistance can affect amphibian disease outcome. Each topic covered describes the amphibian immune system and its modulation by critical biotic and abiotic factors (e.g. stress, hormone manipulation, infection, temperature and dehydration). Moreover, the papers in the issue help to identify and describe which elements of the amphibian immune system contribute to amphibian success in a changing environment. In this way, the theme issue adds novelty to the disease ecology, ecoimmunology and conservation biology fields and offers meaningful insights for future directions.

## 2. Amphibian immune system

Although many aspects of the amphibian immune system have been described in the last decades, most studies have been dedicated to the *Xenopus* genus [34]. In this issue, [35] describes the current knowledge about the amphibian immune system more widely. The authors describe molecular and cellular characteristics, tissue composition and function in anurans, urodeles and legless caecilians [35]. In addition, they point to several immune aspects related to innate and adaptive responses in amphibians, which are better characterized in *Xenopus*, axolotl and the giant salamander. The authors also identify areas where the lack of knowledge is particularly critical for a better understanding of infectious diseases such as chytridiomycosis. They also warn about blind reliance on transcriptomics without a reliable genome reference for rapidly evolving immune genes and without support of functional assays and kinetics of immune responses. Bringing detailed information about amphibian macrophage-lineage cells, [36] demonstrated that *Xenopus laevis* interleukin-34 (IL-34) macrophages and FLT3L dendritic cells share many similarities with colony-stimulating factor-1 (CSF1) macrophages, including transcriptional profiles and functional capacities. The authors also report that the IL34 macrophages and FLT3L dendritic cells have greater MHC class I, but not class II, surface expression, and are better at eliciting mixed leucocyte responses *in vitro* and generating *in vivo* memory immune responses against *Mycobacterium marinum* compared with *X. laevis* CSF1 macrophages [36].

To determine the main effects of the biotic and abiotic influences on the amphibian immune system under natural or laboratory conditions, different wild/free-living amphibian species have been used [16,27,29,33,37–41]. However, the toolbox for measuring the immune system aspects in free-living amphibian species remains limited. In this theme issue, you will find immune variables that can be measured in amphibian species. To measure cellular immunity in the blood, one can use the number of circulating white blood cells (total leucocyte count), the neutrophil to lymphocyte ratio and the phagocytosis activity of blood cells (mainly monocytes and neutrophils). Also, the inflammatory response and wound-healing processes are *in vivo* cellular aspects broadly used to measure innate immunity in many contexts in wild and captive amphibians [28,30,41–43]. Likewise, for non-cellular parameters of the immune system, the plasma bacterial killing ability, a measure that determines the *in vitro* plasma protein's ability to kill a pathogen [44], has been used in several studies [27,30,39,45]. In this issue, highlighting an important aspect of the adaptive immune response, [46] describes ontogenetic differences in IgY levels by showing that post-metamorphic frogs displayed higher IgY antibody levels than tadpoles and metamorphic frogs.

It is worth mentioning that in amphibians, the resident skin microbiota plays an antimicrobial role, helping to eliminate pathogens such as the *Bd* fungus and the ranavirus [47–51]. This theme issue adds to evidence that the microbiome composition is an essential component of the immune system in tadpoles and adult anurans (*X. laevis* and *Pseudacris regilla*) and salamanders (*Notophthalmus viridescens*) [52–54]. Specifically, [52] demonstrates that experimental reductions of the microbiome, by using antimicrobial treatments, during embryonic and larval stages reduced microbial richness and diversity and altered community composition in tadpoles (*X. laevis*) prior to metamorphosis, which could lead to increased disease susceptibility in amphibian tadpoles and adults. However, [52] found no evidence of increased susceptibility for *Bd* in this study. However, the authors propose that developing a gnotobiotic (germ-free) amphibian model system could be a handy tool for future immunological investigations. In addition, inducing resistance against emerging pathogens through prophylaxis is an exciting management strategy that may impact pathogens and their host-associated microbiome [55]. Accordingly, [54] provides evidence that treatment with an increased prophylactic concentration of fungal metabolic products and exposure duration are associated with significant increases in proportions of *Bd*-inhibitory host-associated bacterial taxa. These results suggest a protective prophylactic-induced shift toward microbiome members that are antagonistic to *Bd* and imply that exposure to a pathogen (*Bd*) alters the microbiome to better cope with subsequent encounters with the same pathogen (*Bd*).

## 3. Stress-related immunomodulation

Stress-driven immunomodulation has gained more attention in ectothermic vertebrates in the last decades [25,27,39,56–62], with implications on the stress-induced protective and harmful effects on several immune functions. As observed in other vertebrates, the amphibian immune system is subjected to stressors and its endocrine mediators, including glucocorticoid-induced immunomodulation [27,28,31,32,63,64]. Stress-induced immunomodulation has been described when animals are facing environmental changes (e.g. temperature variation, dehydration and pollution) [13,15,65–68] and well-established stress-induced protocols (e.g. restraint and captivity maintenance) [27,29,69–71]. Interestingly, although these aforementioned stressors are distinct, their impact on amphibian immunity is generally favourable when associated with short-term stressors [16,27,30,60], contrasting with harmful effects under long-term stress conditions [28–30]. However, complex stress-induced immunomodulation is described with increased, decreased and/or no stress impacts on anuran immunity [27,28,30,32,45].

Several papers in this issue describe stress-induced immunomodulation and causal effects of experimental increases in corticosterone on anurans. A review, [72], summarizes effects of heat and dehydration on amphibian immunity, highlighting the importance of these two stressors for amphibian survival. Studies suggest that heat and desiccation stress can activate the hypothalamus–pituitary–interrenal axis, resulting in increased corticosterone plasma levels, with possible immune suppression of some innate and lymphocyte-mediated responses. In addition, increased temperatures can alter microbial communities in amphibian skin and gut, resulting in potential dysbiosis that fosters reduced resistance to pathogens [72].

Regarding corticosterone experimental manipulation, [73] in this special issue shows that increase in plasma corticosterone levels by transdermal application for 48 days induced faster wound-healing when compared with controls in bullfrogs (*Lithobates catesbeianus*), pointing to the immune-enhancing effect of acute daily increases in corticosterone. The authors also show that frogs implanted with corticosterone (1 cm silastic tube filled with powdered corticosterone for 48 to 61 days) tended to heal more slowly than controls (with empty silastic tube), suggesting that the chronic increase in corticosterone can show immunosuppressive effects in these animals [73]. In addition, [53] tested the hypothesis that short- and long-term corticosterone treatment modulates the immune system in a salamander model (*N. viridescens*). The authors found decreased plasma bacterial killing ability and melanomacrophage centre and lower skin microbiome after long-term treatment (26 days), compared with short-term treatment (5 days), but these changes were not associated with corticosterone treatment. These results demonstrate plasma corticosterone increases may not result in immune changes in the eastern newt, at least in the investigated immune aspects [53].

## 4. Amphibian infectious diseases

Several infectious diseases affect amphibians [74]; some of the most common include (i) red leg syndrome: bacterial septicaemia commonly caused by *Aeromonas hydrophila*, resulting in erythema (redness) and oedema (swelling) on the abdomen and ventral part of the posterior members, which may progress to other parts of the body [75,76]. Infected animals may also develop ulcers, open wounds and internal organ damage. In severe cases, it can ultimately lead to death [75,76]. (ii) Chytridiomycosis: caused by the chytrid fungus *B. dendrobatidis* (*Bd*) or *Batrachochytrium salamandrivorans* (*Bsal*), which affects the skin of amphibians, interfering with their respiration and osmoregulation [51,77,78]. Infected animals can develop skin ulcers, increased skin sloughing, lethargy and loss of appetite. *Bd* and *Bsal* have been implicated in significant declines and extinctions of amphibian populations worldwide [2,79]. (iii) Ranavirosis: caused by double-stranded DNA viruses belonging to the family *Iridoviridae* with various symptoms in amphibians, including haemorrhaging, skin ulcers, tissue necrosis and internal organ damage [80,81]. It can cause mass die-offs of infected amphibians and has been identified as a significant threat to populations in many parts of the world [82,83]. (iv) Parasitic infections: amphibians can be affected by a range of internal and external parasites, such as nematodes (roundworms), trematodes (flatworms), mites and ticks [84,85].

Pathogens/parasites may alter host physiology to enhance pathogen/parasite proliferation, survival and transmission [85]. Complex relationships between pathogen/parasite species and their interactions with each other and their hosts might impact infectious disease outcomes [86,87]. In this theme issue, [88] reviews the importance of tolerance and resistance against the fungi *Bd* and *Bsal*, the causative agents of chytridiomycosis. Tolerance measures the ability of an organism to limit the detrimental effects caused by a given infection, protecting the host without harming the pathogen [89]. While resistance refers to the ability to limit the intensity of that infection, protecting the host at the cost of the pathogen [90]. In accordance, [88] highlights that infection tolerance has important implications for pathogen spread and maintenance, drives some species to decline, and contributes to the dilution of natural selection for tolerance and resistance. Similarly, considering the importance of tolerance and resistance, [46] investigates how changes in host immunity through ontogeny can influence interactions among co-infecting parasite species in amphibians. The researchers exposed Cuban treefrogs (*Osteopilus septentrionalis*) to *Bd* fungus and a nematode (*Aplectana hamatospicula*) at different life stages: tadpole, metamorphic and post-metamorphic. The results showed ontogenetic differences in IgY levels and cellular immunity but no evidence of facilitative interactions between co-infecting parasites. However, the *Bd* fungus decreased immunity in metamorphic frogs, making them less resistant and tolerant to infection than other life stages. These findings suggest that immune changes through ontogeny can alter host responses to parasite exposure [46].

Another crucial factor impacting the outcome of host–pathogen interactions is the host microbial communities [91–93]. The adaptive microbiome hypothesis states that exposure to a pathogen alters an organism's microbiome to better cope with subsequent pathogen encounters [94–96]. To assess this hypothesis for chytridiomycosis, [54] investigates the effects of a *Bd* metabolite-based prophylactic inoculation on the host microbiome composition in larval *P. regilla.* Their results suggest a protective prophylactic-induced shift toward microbiome members antagonistic to *Bd* [54]. Furthermore, diet composition significantly impacts the diversity and function of host-associated gut microbial communities [97,98]. For example, high-quality diets can improve the host's nutrient uptake and alter the metabolites used and produced by the host and its microbial communities [99,100]. In this theme issue, [101] investigates how increasing salinization in freshwaters by road de-icing salt runoff affects gut bacterial assembly, host physiology and responses to ranavirus exposure in larval wood frogs (*Rana sylvatica*). The authors found that elevating salinity and supplementing a basic larval diet with algae increased larval growth but also increased ranavirus loads. However, larvae given algae did not exhibit elevated corticosterone levels, accelerated development or weight loss post-infection, whereas larvae fed a basic diet did. The authors suggest that algal supplementation may reduce stress responses to infection by regulating host metabolism and endocrine function [101].

In addition, the emergence of novel pathogens in naive multi-host communities can have differential impacts on species, with some acting as reservoirs and others amplifying transmission [102,103]. However, characterizing the roles of different species in wildlife communities during infectious disease emergence is challenging as these events are often unpredictable. How species-specific attributes influence exposure, probability of infection, and pathogen intensity during the fungal pathogen (*Bd*) emergence was the central question of [104] in this issue. The authors used field-collected data and found that some hosts disproportionately contributed to transmission dynamics [104]. This information is particularly important when considering reintroducing amphibians back into their original communities. If supersensitive hosts are reintroduced and are unable to overcome infections, this can have a detrimental impact on the success of conservation programmes. By identifying key species responsible for disease transmission, conservationists can better understand and mitigate the risk of disease transmission, thereby improving the chances of successful reintroduction programs.

## 5. Ecoimmunology

Ecoimmunology is a field of study that investigates the interactions between an organism's immune system and its environment. It is an interdisciplinary field integrating concepts and methods from ecology, evolution and immunology [105,106]. Ecoimmunology seeks to understand how the immune system of an organism adapts to environmental conditions and stressors, such as temperature, food availability and exposure to pathogens [107,108]. It also examines how immune responses influence the fitness and survival of individuals, populations and species [109,110]. Research in ecoimmunology has revealed that environmental conditions and stressors can significantly impact immune function, including changes in the number and type of immune cells produced, the speed and strength of immune responses and the susceptibility of individuals to diseases [21,32,62,111].

In this theme issue, [112] investigates the links between changes in body mass of captive cane toads (*Rhinella marina*) and their performance in immune assays. Toads that lost weight over three months had a higher phagocytic ability of whole blood owing to increased circulating levels of phagocytic cells. Other measures of immune performance were not correlated with mass change. Considering the challenges invasive species face as they expand into novel environments with substantial seasonal changes in food availability, energy restrictions might shift their immune function toward more economical and general avenues of combating pathogens [112]. Another paper of this issue, [113] investigates a native Brazilian toad species (*Rhinella icterica*), to establish a positive correlation between testosterone, corticosterone and immune function in the field, and a positive correlation between testosterone and immune function in captive male toads, suggesting these steroids show an immunostimulatory effect during the reproduction in anurans [113].

Considering that amphibians face many threats, including habitat degradation, introduced species, pollutants, emerging diseases, unpredictable temperature changes and rainfall, [72] reviews amphibians' response to some natural stressors. The authors demonstrate that in addition to possibly suppressing some innate and lymphocyte-mediated responses, elevated temperatures can alter amphibian skin and gut microbial communities, resulting in possible dysbiosis that fosters reduced resistance to pathogens. Thus, extreme heat and drought stresses due to climate change might increase the vulnerability of amphibians to diseases such as chytridiomycosis and ranavirus outbreaks [72]. The authors emphasize the importance of understanding host–pathogen physiology for determining how amphibian populations deal with stressful situations and cope with diseases in a changing world.

## 6. Conservation biology

The conservation physiology field comprises diverse physiological topics, such as immune and endocrine systems and nutritional traits, to understand organismal and population responses to environmental change, stressors and disease resistance [21,114]. Among vertebrates, amphibians are the most threatened and declining group, with more than 40% of amphibian species being recognized as endangered [115].

Amphibians have a complex life cycle and are predicted to be among the vertebrate taxa most affected by climate change, pathogens and other stressors [7,115,116]. In particular, climate change is expected to promote changes in wetland temperatures, hydroperiod or drought regimes, among others, with amphibians being a great model in the context of monitoring and predicting the health, persistence, and distribution of free-living populations in the face of environmental changes [7,117]. Conservation efforts often focus on preventing and managing the spread of diseases and addressing other factors contributing to amphibian declines. Pathogens like *Bd* and *Bsal* are relatively new; however, even for those known for longer, such as bacteria and ranaviruses, much remains unknown about their distribution and impacts on amphibian populations.

The papers in this theme issue provide knowledge, approaches and techniques that can address the impacts of changing landscapes, experimental manipulation and infectious disease across amphibian individual and populational scales. This theme issue brings also an array of ecoimmunological tools for amphibians, such as immune measures (e.g. white blood cell profile, plasma bacterial killing ability and phagocytosis assay), stress-induced hormonal and immune modulation, microbiome composition and function, and disease resistance. These tools can be used in wildlife and experimental conditions to manage emerging pathogens, and monitor and predict disease susceptibility in amphibian populations, helping to understand amphibian survival in a changing world. Moreover, the papers in this issue bring insights into the importance of understanding the amphibian immune response (at larval and adult stages) to the diseases to understand better the mechanisms that drive differences among populations and species [36,46,52,54,88,104], which could help to predict amphibian conservation strategies.

Preventing the spread of diseases will require careful management from conservation organizations and government agencies, including increased surveillance of wild populations, restrictions on the import and trade of amphibians, assessment of water and environmental quality, and effective quarantine measures for infected individuals. Understanding the relationship between immune function and the environment is essential for conservation efforts, as environmental stressors and habitat degradation can negatively impact immune function, leaving populations more vulnerable to diseases and other threats. By studying ecoimmunology, researchers can gain insight into how to promote the health and resilience of individuals and populations in changing and challenging environments. In addition, by understanding and managing the factors that threaten biodiversity, conservation biologists can help ensure a sustainable future for both human society and the natural world.

## 7. Conclusions, future directions, and limitations

Despite the fact that amphibians have been used as a model system for studying the climate change effects on the immune system and disease outcomes, our capacity to establish expectations across different environmental conditions is limited. Moreover, attempts to understand the integrated immune and endocrine responses and disease susceptibility following environmental changes are scarce. The papers in this theme issue underscore that amphibians have several cellular and non-cellular immune aspects that can be used to investigate wild and captive populations in many biotic and abiotic conditions. They also underline the importance of ontogeny, emphasizing the urge to understand the biotic and abiotic effects on immunity in amphibians at different life stages. The present papers suggest that stress-driven immunomodulation in distinct amphibian species is complex and bring essential insights into understanding amphibians' physiology, ecology, evolution and conservation. There is also research covering several aspects of the amphibian immune system associated with infectious diseases, which will advance comparative investigations on amphibian immunity in the proposed topics. Since investigated variables were tested and validated for multiple species, standardizing the methods for analysing amphibian immunity across species will help to achieve a more integrative approach for future studies. However, several knowledge gaps remain, and exploring future directions while considering the limitations is essential for advancing our understanding of these intricate relationships. Below we highlight the potential future directions and acknowledge the limitations that researchers might encounter when investigating amphibian ecoimmunology, stress modulation, and disease ecology.

### (a) Future directions

#### (i) Investigating the role of the microbiome

The microbiome influences immune system function and stress responses in various organisms. Understanding the composition and function of the amphibian microbiome and its interactions with the host's immune system could provide valuable insights into disease susceptibility and stress modulation.

#### (ii) Integrating multi-omics approaches

Combining genomic, transcriptomic, proteomic and metabolomic data can enhance our understanding of the underlying mechanisms involved in stress responses, immune function, and disease susceptibility in amphibians. Integrating multi-omics approaches will enable a comprehensive assessment of the complex interactions between genes, environment and pathogens.

#### (iii) Functional and longitudinal studies

An important aspect of stress and immune responses against pathogens is their sequential development across the different organs within an organism and over time. While multi-omics approaches are powerful, they are fully adequate to capture the dynamic response of immune cells in space and time, their expansion, contraction, migration, homing, and effector function (cell killing, phagocytosis, etc.). While functional studies are challenging in non-model species (e.g. lack of reagents such as antibodies), new technology such as single-cell transcriptomics provides a powerful new opportunity to advance these types of studies.

#### (iv) Assessing the effects of anthropogenic stressors

Amphibians face numerous anthropogenic stressors, such as habitat loss, pollution, and climate change. Future research should focus on examining how these environmental stressors impact amphibian immune function, stress responses, and disease dynamics. Identifying the mechanisms by which anthropogenic stressors compromise amphibian health will aid in developing effective conservation strategies.

#### (v) Examining trade-offs in immune investment

Investigating the trade-offs between immune function and other life-history traits, such as reproductive investment and growth, is crucial. Understanding how amphibians allocate limited resources to different physiological processes will provide insights into the costs and benefits of immune responses and their ecological implications.

### (b) Limitations

#### (i) Sample size and statistical power

Conducting studies with large sample sizes is challenging in the field of amphibian ecoimmunology owing to logistical constraints, limited funding, and the decline of amphibian populations and risk of disturbance of small populations (e.g. reproduction). Small sample sizes limit the statistical power and generalizability of research findings.

#### (ii) Environmental variability

Amphibians inhabit very diverse environments with a wide variety of ecological conditions. The dynamic nature of these environments/microenvironments can introduce confounding factors and make it difficult to discern the specific effects of stressors and diseases on amphibian immune function.

#### (iii) Pathogen diversity and interactions

Amphibians are susceptible to numerous pathogens, including viruses, fungi, bacteria and parasites. The interactions between multiple pathogens and their impacts on amphibian health are complex and not yet fully understood. Investigating the interactions and co-infections among pathogens will be crucial to unravel their specific and combined effects on amphibian immune responses.

#### (iv) Long-term monitoring

Long-term studies that track individual amphibians across their lifespan are needed to understand the cumulative effects of stress, disease, and environmental changes. However, such studies require substantial resources and long-term commitment, making them challenging to conduct.

## Data Availability

This article has no additional data.

## References

[1] Collins JP. 2010 Amphibian decline and extinction: what we know and what we need to learn. Dis. Aquat. Organ. **92**, 93-99. (10.3354/dao02307) 21268970

[2] Scheele BC et al. 2019 Amphibian fungal panzootic causes catastrophic and ongoing loss of biodiversity. Science **363**, 1459-1463. (10.1126/science.aav0379) 30923224

[3] Rollins-Smith LA, Ramsey JP, Pask JD, Reinert LK, Woodhams DC. 2011 Amphibian immune defenses against chytridiomycosis: impacts of changing environments. Integr. Comp. Biol. **51**, 552-562. (10.1093/icb/icr095) 21816807

[4] Carey C, Alexander MA. 2003 Climate change and amphibian declines: is there a link? Divers. Distrib. **9**, 111-121. (10.1046/j.1472-4642.2003.00011.x)

[5] Mendelson III JR et al. 2006 Biodiversity. Confronting amphibian declines and extinctions. Science **313**, 48. (10.1126/science.1128396) 16825553

[6] Rohr JR, Raffel TR, Romansic JM, McCallum H, Hudson PJ. 2008 Evaluating the links between climate, disease spread, and amphibian declines. Proc. Natl Acad. Sci. USA **105**, 17 436-17 441. (10.1073/pnas.0806368105) PMC2582253 18987318

[7] Rollins-Smith LA. 2017 Amphibian immunity–stress, disease, and climate change. Dev. Comp. Immunol. **66**, 111-119. (10.1016/j.dci.2016.07.002) 27387153

[8] Janin A, Léna JP, Deblois S, Joly P. 2012 Use of stress-hormone levels and habitat selection to assess functional connectivity of a landscape for an amphibian. Conserv. Biol. **26**, 923-931. (10.1111/j.1523-1739.2012.01910.x) 22891816

[9] Narayan EJ, Cockrem JF, Hero JM. 2012 Effects of temperature on urinary corticosterone metabolite responses to short-term capture and handling stress in the cane toad (*Rhinella marina*). Gen. Comp. Endocrinol. **178**, 301-305. (10.1016/j.ygcen.2012.06.014) 22728158

[10] Raffel TR, Rohr JR, Kiesecker JM, Hudson PJ. 2006 Negative effects of changing temperature on amphibian immunity under field conditions. Funct. Ecol. **20**, 819-828. (10.1111/j.1365-2435.2006.01159.x)

[11] Gabor CR, Fisher MC, Bosch J. 2015 Elevated corticosterone levels and changes in amphibian behavior are associated with *Batrachochytrium dendrobatidis* (*Bd*) infection and *Bd* lineage. PLoS ONE **10**, e0122685. (10.1371/journal.pone.0122685) 25893675 PMC4404099

[12] Kolby JE. 2018 Amphibia: global amphibian declines caused by an emerging infectious disease and inadequate immune responses. In Advances in comparative immunology (ed. EL Cooper), pp. 981-990. Cham, Switzerland: Springer International Publishing.

[13] Lima AS, de Figueredo AC, Floreste FR, Garcia Neto PG, Gomes FR, Titon SCM. 2022 Temperature extreme events decrease endocrine and immune reactive scope in bullfrogs (*Lithobates catesbeianus*). Integr. Comp. Biol. **62**, 1671-1682. (10.1093/icb/icac105) 35771987

[14] Ribas L et al. 2009 Expression profiling the temperature-dependent amphibian response to infection by *Batrachochytrium dendrobatidis*. PLoS ONE **4**, e8408. (10.1371/journal.pone.0008408) 20027316 PMC2794374

[15] Barsotti AMG, Titon Junior B, Titon SCM, Gomes FR. 2019 Dehydration as a stressor in toads (*Rhinella ornata*). J. Exp. Zool. A Ecol. Integr. Physiol. **331**, 168-174. (10.1002/jez.2250) 30569667

[16] Barsotti AMG, Madelaire CB, Wagener C, Titon B, Measey J, Gomes FR. 2021 Challenges of a novel range: water balance, stress, and immunity in an invasive toad. Comp. Biochem. Physiol. A Mol. Integr. Physiol. **253**, 110870. (10.1016/j.cbpa.2020.110870) 33321177

[17] Novarro AJ, Gabor CR, Goff CB, Mezebish TD, Thompson LM, Grayson KL. 2018 Physiological responses to elevated temperature across the geographic range of a terrestrial salamander. J. Exp. Biol. **221**, jeb178236. (10.1242/jeb.178236) 30072387

[18] Peterson JD, Steffen JE, Reinert LK, Cobine PA, Appel A, Rollins-Smith LA, Mendonça MT. 2013 Host stress response is important for the pathogenesis of the deadly amphibian disease, chytridiomycosis, in *Litoria caerulea*. PLoS ONE **8**, e62146. (10.1371/journal.pone.0062146) 23630628 PMC3632538

[19] Kindermann C, Narayan EJ, Hero JM. 2012 Urinary corticosterone metabolites and chytridiomycosis disease prevalence in a free-living population of male Stony Creek frogs (*Litoria wilcoxii*). Comp. Biochem. Physiol. A Mol. Integr. Physiol. **162**, 171-176. (10.1016/j.cbpa.2012.02.018) 22387450

[20] Kindermann C, Narayan EJ, Hero JM. 2017 Does physiological response to disease incur cost to reproductive ecology in a sexually dichromatic amphibian species? Comp. Biochem. Physiol. A Mol. Integr. Physiol. **203**, 220-226. (10.1016/j.cbpa.2016.09.019) 27712921

[21] Ohmer MEB et al. 2021 Applied ecoimmunology: using immunological tools to improve conservation efforts in a changing world. Conserv. Physiol. **9**, coab074. (10.1093/conphys/coab074) 34512994 PMC8422949

[22] Gabor CR, Fisher MC, Bosch J. 2013 A non-invasive stress assay shows that tadpole populations infected with *Batrachochytrium dendrobatidis* have elevated corticosterone levels. PLoS ONE **8**, e56054. (10.1371/journal.pone.0056054) 23418508 PMC3572145

[23] Dhabhar FS. 2018 The short-term stress response – Mother nature's mechanism for enhancing protection and performance under conditions of threat, challenge, and opportunity. Front. Neuroendocrinol. **49**, 175-192. (10.1016/j.yfrne.2018.03.004) 29596867 PMC5964013

[24] Dhabhar FS. 2014 Effects of stress on immune function: the good, the bad, and the beautiful. Immunol. Res. **58**, 193-210. (10.1007/s12026-014-8517-0) 24798553

[25] Demas GE, Adamo SA, French SS. 2011 Neuroendocrine-immune crosstalk in vertebrates and invertebrates: implications for host defence. Funct. Ecol. **25**, 29-39. (10.1111/j.1365-2435.2010.01738.x)

[26] Martin LB, Kernbach ME, Unnasch TR. 2019 Distinct effects of acute versus chronic corticosterone exposure on zebra finch responses to West Nile virus. Conserv. Physiol. **7**, coz094. (10.1093/conphys/coz094) 31824675 PMC6894510

[27] Titon SCM, Titon Junior B, Gomes FR, Assis VR. 2021 Short-term stressors and corticosterone effects on immunity in male toads (*Rhinella icterica*): a neuroimmune-endocrine approach. Brain Behav. Immun. Health **13**, 100230. (10.1016/j.bbih.2021.100230) 34589745 PMC8474493

[28] Titon SCM, Titon Junior B, Barsotti AMG, Gomes FR, Assis VR. 2019 Time-related immunomodulation by stressors and corticosterone transdermal application in toads. PLoS ONE **14**, e0222856. (10.1371/journal.pone.0222856) 31539413 PMC6754171

[29] Titon SCM, Titon Junior B, Assis VR, Kinker GS, Fernandes PACM, Gomes FR. 2018 Interplay among steroids, body condition and immunity in response to long-term captivity in toads. Scient. Rep. **8**, 17168. (10.1038/s41598-018-35495-0) PMC6249311 30464319

[30] Assis VR, Titon SCM, Barsotti AMG, Titon Junior B, Gomes FR. 2015 Effects of acute restraint stress, prolonged captivity stress and transdermal corticosterone application on immunocompetence and plasma levels of corticosterone on the cururu toad (*Rhinella icterica*). PLoS ONE **10**, e0121005. (10.1371/journal.pone.0121005) 25831055 PMC4382218

[31] Assis VR, Titon SCM, Queiroz-Hazarbassanov NGT, Massoco CO, Gomes FR. 2017 Corticosterone transdermal application in toads (*Rhinella icterica*): effects on cellular and humoral immunity and steroid plasma levels. J. Exp. Zool. A Ecol. Integr. Physiol. **327**, 200-213. (10.1002/jez.2093) 29356458

[32] Gomes FR, Madelaire CB, Moretti EH, Titon SCM, Assis VR. 2022 Immunoendocrinology and ecoimmunology in Brazilian anurans. Integr. Comp. Biol. **62**, 1654-1670. (10.1093/icb/icac014) 35411921

[33] Hopkins WA, DuRant SE. 2011 Innate immunity and stress physiology of eastern hellbenders (*Cryptobranchus alleganiensis*) from two stream reaches with differing habitat quality. Gen. Comp. Endocrinol. **174**, 107-115. (10.1016/j.ygcen.2011.08.006) 21872597

[34] Robert J, Ohta Y. 2009 Comparative and developmental study of the immune system in *Xenopus*. Dev. Dyn. **238**, 1249-1270. (10.1002/dvdy.21891) 19253402 PMC2892269

[35] Ruiz VL, Robert J. 2023 The amphibian immune system. Phil. Trans. R. Soc. B **378**, 20220123. (10.1098/rstb.2022.0123) 37305914 PMC10258673

[36] Hossainey MRH, Hauser KA, Garvey CN, Kalia N, Garvey JM, Grayfer L. 2023 A perspective into the relationships between amphibian (*Xenopus laevis*) myeloid cell subsets. Phil. Trans. R. Soc. B **378**, 20220124. (10.1098/rstb.2022.0124) 37305910 PMC10258660

[37] Jessop TS, Lane M, Wilson RS, Narayan EJ. 2018 Testing for short- and long-term thermal plasticity in corticosterone responses of an ectothermic vertebrate. Physiol. Biochem. Zool. **91**, 967-975. (10.1086/698664) 29863953

[38] Gardner ST, Assis VR, Zhao H, Gomes FR, Peatman E, Mendonça MT. 2018 Differential gene expression to an LPS challenge in relation to exogenous corticosterone in the invasive cane toad (*Rhinella marina*). Dev. Comp. Immunol. **88**, 114-123. (10.1016/j.dci.2018.07.016) 30030104

[39] Assis VR, Gardner ST, Smith KM, Gomes FR, Mendonça MT. 2020 Stress and immunity: field comparisons among populations of invasive cane toads in Florida. J. Exp. Zool. A Ecol. Integr. Physiol. **333**, 779-791. (10.1002/jez.2389) 32488987

[40] Barsotti AMG, de Assis VR, Titon SCM, Titon Junior B, da Silva Ferreira ZF, Gomes FR. 2017 ACTH modulation on corticosterone, melatonin, testosterone and innate immune response in the tree frog *Hypsiboas faber*. Comp. Biochem. Physiol. A Mol. Integr. Physiol. **204**, 177-184. (10.1016/j.cbpa.2016.12.002) 27923708

[41] Thomas JR, Woodley SK. 2015 Treatment with corticosterone delays cutaneous wound healing in male and female salamanders. Gen. Comp. Endocrinol. **216**, 33-38. (10.1016/j.ygcen.2015.04.013) 25913258

[42] Brown GP, Shilton CM, Shine R. 2011 Measuring amphibian immunocompetence: validation of the phytohemagglutinin skin-swelling assay in the cane toad, *Rhinella marina*. Methods Ecol. Evol. **2**, 341-348. (10.1111/j.2041-210X.2011.00090.x)

[43] Madelaire CB, Cassettari BdO, Gomes FR. 2019 Immunomodulation by testosterone and corticosterone in toads: experimental evidences from transdermal application. Gen. Comp. Endocrinol. **273**, 227-235. (10.1016/j.ygcen.2018.09.005) 30195026

[44] Assis VR, Titon SCM, Barsotti AMG, Spira B, Gomes FR. 2013 Antimicrobial capacity of plasma from anurans of the Atlantic Forest. South Am. J. Herpetol. **8**, 155-160. (10.2994/sajh-d-13-00007.1)

[45] Graham SP, Kelehear C, Brown GP, Shine R. 2012 Corticosterone–immune interactions during captive stress in invading Australian cane toads (*Rhinella marina*). Horm. Behav. **62**, 146-153. (10.1016/j.yhbeh.2012.06.001) 22713726

[46] Ramsay C, Rohr JR. 2023 Ontogeny of immunity and potential implications for co-infection. Phil. Trans. R. Soc. B **378**, 20220127. (10.1098/rstb.2022.0127) 37305918 PMC10258665

[47] Grogan LF, Robert J, Berger L, Skerratt LF, Scheele BC, Castley JG, Newell DA, McCallum HI. 2018 Review of the amphibian immune response to chytridiomycosis, and future directions. Front. Immunol. **9**, 2536. (10.3389/fimmu.2018.02536) 30473694 PMC6237969

[48] Holden WM et al. 2015 Skin bacteria provide early protection for newly metamorphosed southern leopard frogs (*Rana sphenocephala*) against the frog-killing fungus, *Batrachochytrium dendrobatidis*. Biol. Conserv. **187**, 91-102. (10.1016/j.biocon.2015.04.007)

[49] Jiménez RR, Sommer S. 2017 The amphibian microbiome: natural range of variation, pathogenic dysbiosis, and role in conservation. Biodivers. Conserv. **26**, 763-786. (10.1007/s10531-016-1272-x)

[50] Campbell LJ, Garner TWJ, Hopkins K, Griffiths AGF, Harrison XA. 2019 Outbreaks of an emerging viral disease covary with differences in the composition of the skin microbiome of a wild United Kingdom amphibian. Front. Microbiol. **10**, 1245. (10.3389/fmicb.2019.01245) 31281291 PMC6597677

[51] Grogan LF, Humphries JE, Robert J, Lanctôt CM, Nock CJ, Newell DA, McCallum HI. 2020 Immunological aspects of chytridiomycosis. J. Fungi **6**, 234. (10.3390/jof6040234) PMC7712659 33086692

[52] Miller AJ, Gass J, Jo MC, Bishop L, Petereit J, Woodhams DC, Voyles J. 2023 Towards the generation of gnotobiotic larvae as a tool to investigate the influence of the microbiome on the development of the amphibian immune system. Phil. Trans. R. Soc. B **378**, 20220125. (10.1098/rstb.2022.0125) 37305911 PMC10258664

[53] Pereira KE, Bletz MC, McCartney JA, Woodhams DC, Woodley SK. 2023 Effects of exogenous elevation of corticosterone on immunity and the skin microbiome of eastern newts (*Notophthalmus viridescens*). Phil. Trans. R. Soc. B **378**, 20220120. (10.1098/rstb.2022.0120) 37305906 PMC10258667

[54] Siomko SA, Greenspan SE, Barnett KM, Neely WJ, Chtarbanova S, Woodhams DC, McMahon TA, Becker CG. 2023 Selection of an anti-pathogen skin microbiome following prophylaxis treatment in an amphibian model system. Phil. Trans. R. Soc. B **378**, 20220126. (10.1098/rstb.2022.0126) 37305917 PMC10258671

[55] Cross ML, Buddle BM, Aldwell FE. 2007 The potential of oral vaccines for disease control in wildlife species. Vet. J. **174**, 472-480. (10.1016/j.tvjl.2006.10.005) 17113798

[56] Lazado CC, Gesto M, Madsen L, Jokumsen A. 2018 Interplay between daily rhythmic serum-mediated bacterial killing activity and immune defence factors in rainbow trout (*Oncorhynchus mykiss*). Fish Shellfish Immunol. **72**, 418-425. (10.1016/j.fsi.2017.11.025) 29146445

[57] Neuman-Lee LA, French SS. 2017 Endocrine-reproductive-immune interactions in female and male Galápagos marine iguanas. Horm. Behav. **88**, 60-69. (10.1016/j.yhbeh.2016.10.017) 27818221

[58] Moeller KT, Demare G, Davies S, DeNardo DF. 2017 Dehydration enhances multiple physiological defense mechanisms in a desert lizard, *Heloderma suspectum*. J. Exp. Biol. **220**, 2166-2174. (10.1242/jeb.150367) 28432151

[59] Neuman-Lee LA, Bobby Fokidis H, Spence AR, Van der Walt M, Smith GD, Durham S, French SS. 2015 Food restriction and chronic stress alter energy use and affect immunity in an infrequent feeder. Funct. Ecol. **29**, 1453-1462. (10.1111/1365-2435.12457)

[60] Seddon RJ, Klukowski M. 2012 Influence of stressor duration on leukocyte and hormonal responses in male southeastern five-lined skinks (*Plestiodon inexpectatus*). J. Exp. Zool. A Ecol. Genet. Physiol. **317**, 499-510. (10.1002/jez.1742) 22791614

[61] Titon Jr B, Titon SCM, Assis VR, Barsotti AMG, Vasconcelos-Teixeira R, Fernandes PACM, Gomes FR. 2021 LPS-induced immunomodulation and hormonal variation over time in toads. J. Exp. Zool. A Ecol. Integr. Physiol. **335**, 541-551. (10.1002/jez.2474) 34018702

[62] Assis VR, Titon SCM, Voyles J. 2022 Ecoimmunology: what unconventional organisms tell us after two decades. Integr. Comp. Biol. **62**, 1528-1535. (10.1093/icb/icac148) 36250609

[63] Billig ST, Weber RN, Zimmerman LM, Wilcoxen TE. 2020 Effects of elevated corticosterone on humoral innate and antibody-mediated immunity in southern leopard frog (*Lithobates sphenocephalus*) tadpoles. J. Exp. Zool. A Ecol. Integr. Physiol. **333**, 756-766. (10.1002/jez.2406) 32798287

[64] Falso PG, Noble CA, Diaz JM, Hayes TB. 2015 The effect of long-term corticosterone treatment on blood cell differentials and function in laboratory and wild-caught amphibian models. Gen. Comp. Endocrinol. **212**, 73-83. (10.1016/j.ygcen.2015.01.003) 25616196

[65] Bókony V, Ujhegyi N, Hamow KÁ, Bosch J, Thumsová B, Vörös J, Aspbury AS, Gabor CR. 2021 Stressed tadpoles mount more efficient glucocorticoid negative feedback in anthropogenic habitats due to phenotypic plasticity. Sci. Total Environ. **753**, 141896. (10.1016/j.scitotenv.2020.141896) 32889314

[66] Clay TA, Steffen MA, Treglia ML, Torres CD, Trujano-Alvarez AL, Bonett RM. 2019 Multiple stressors produce differential transcriptomic patterns in a stream-dwelling salamander. BMC Genomics **20**, 482. (10.1186/s12864-019-5814-y) 31185901 PMC6560913

[67] Brannelly LA, Ohmer MEB, Saenz V, Richards-Zawacki CL. 2019 Effects of hydroperiod on growth, development, survival and immune defences in a temperate amphibian. Funct. Ecol. **33**, 1952-1961. (10.1111/1365-2435.13419)

[68] Burraco P, Gomez-Mestre I. 2016 Physiological stress responses in amphibian larvae to multiple stressors reveal marked anthropogenic effects even below lethal levels. Physiol. Biochem. Zool. **89**, 462-472. (10.1086/688737) 27792531

[69] Narayan EJ, Molinia FC, Cockrem JF, Hero JM. 2011 Changes in urinary testosterone and corticosterone metabolites during short-term confinement with repeated handling in wild male cane toads (*Rhinella marina*). Aust. J. Zool. **59**, 264-269. (10.1071/ZO11070)

[70] Assis VR, Titon SCM, Gomes FR. 2019 Acute stress, steroid plasma levels, and innate immunity in Brazilian toads. Gen. Comp. Endocrinol. **273**, 86-97. (10.1016/j.ygcen.2018.05.008) 29750968

[71] Titon SCM, Titon Junior B, de Figueiredo AC, Floreste FR, Siqueira Lima A, Cyrino JC, Gomes FR. 2022 Plasma steroids and immune measures vary with restraint duration in a toad (*Rhinella icterica*). Gen. Comp. Endocrinol. **318**, 113987. (10.1016/j.ygcen.2022.113987) 35131311

[72] Rollins-Smith LA, Le Sage EH. 2023 Heat stress and amphibian immunity in a time of climate change. Phil. Trans. R. Soc. B **378**, 20220132. (10.1098/rstb.2022.0132) 37305907 PMC10258666

[73] Madelaire CB, Silva DP, Titon SCM, Lamadrid-Feris F, Floreste FR, Titon Jr B, Gomes FR. 2023 Contrasting effects of transdermal and implant corticosterone treatments in the American bullfrog wound healing. Phil. Trans. R. Soc. B **378**, 20220119. (10.1098/rstb.2022.0119) 37305919 PMC10258662

[74] Densmore CL, Green DE. 2007 Diseases of amphibians. ILAR J. **48**, 235-254. (10.1093/ilar.48.3.235) 17592186

[75] Rivas ZP. 2016 *Aeromonas hydrophila* in amphibians: harmless bystander or opportunistic pathogen? Honors undergraduate thesis, University of Central Florida, Orlando, FL. See https://stars.library.ucf.edu/honorstheses/13.

[76] Hill WA, Newman SJ, Craig L, Carter C, Czarra J, Brown JP. 2010 Diagnosis of *Aeromonas hydrophila*, Mycobacterium species, and Batrachochytrium dendrobatidis in an African clawed frog (*Xenopus laevis*). J. Am. Assoc. Lab. Anim. Sci. **49**, 215-220. 20353698 PMC2846011

[77] Voyles J, Berger L, Young S, Speare R, Webb R, Warner J, Rudd D, Campbell R, Skerratt L. 2007 Electrolyte depletion and osmotic imbalance in amphibians with chytridiomycosis. Dis. Aquat. Organ. **77**, 113-118. (10.3354/dao01838) 17972752

[78] Martel A et al. 2013 Batrachochytrium salamandrivorans sp. nov. causes lethal chytridiomycosis in amphibians. Proc. Natl Acad. Sci. USA **110**, 15 325-15 329. (10.1073/pnas.1307356110) PMC3780879 24003137

[79] Berger L et al. 1998 Chytridiomycosis causes amphibian mortality associated with population declines in the rain forests of Australia and Central America. Proc. Natl Acad. Sci. USA **95**, 9031-9036. (10.1073/pnas.95.15.9031) 9671799 PMC21197

[80] Gray M, Miller D, Hoverman J. 2009 Ecology and pathology of amphibian ranaviruses. Dis. Aquat. Organ. **87**, 243-266. (10.3354/dao02138) 20099417

[81] Blaustein A et al. 2018 Effects of emerging infectious diseases on amphibians: a review of experimental studies. Diversity **10**, 81. (10.3390/d10030081)

[82] North AC, Hodgson DJ, Price SJ, Griffiths AGF. 2015 Anthropogenic and ecological drivers of amphibian disease (ranavirosis). PLoS ONE **10**, e0127037. (10.1371/journal.pone.0127037) 26039741 PMC4454639

[83] Teacher AGF, Cunningham AA, Garner TWJ. 2010 Assessing the long-term impact of ranavirus infection in wild common frog populations. Anim. Conserv. **13**, 514-522. (10.1111/j.1469-1795.2010.00373.x)

[84] Johnson PTJ, Hoverman JT. 2012 Parasite diversity and coinfection determine pathogen infection success and host fitness. Proc. Natl Acad. Sci. USA **109**, 9006-9011. (10.1073/pnas.1201790109) 22615371 PMC3384156

[85] Hernandez-Caballero I, Garcia-Longoria L, Gomez-Mestre I, Marzal A. 2022 The adaptive host manipulation hypothesis: parasites modify the behaviour, morphology, and physiology of amphibians. Diversity **14**, 739. (10.3390/d14090739)

[86] Martin LB, Burgan SC, Adelman JS, Gervasi SS. 2016 Host competence: an organismal trait to integrate immunology and epidemiology. Integr. Comp. Biol. **56**, 1225-1237. (10.1093/icb/icw064) 27940614

[87] Becker DJ, Downs CJ, Martin LB. 2019 Multi-scale drivers of immunological variation and consequences for infectious disease dynamics. Integr. Comp. Biol. **59**, 1129-1137. (10.1093/icb/icz138) 31559436

[88] Grogan LF, Mangan MJ, McCallum HI. 2023 Amphibian infection tolerance to chytridiomycosis. Phil. Trans. R. Soc. B **378**, 20220133. (10.1098/rstb.2022.0133) 37305912 PMC10258672

[89] Råberg L, Graham AL, Read AF. 2009 Decomposing health: tolerance and resistance to parasites in animals. Phil. Trans. R. Soc. B **364**, 37-49. (10.1098/rstb.2008.0184) 18926971 PMC2666700

[90] Kutzer MAM, Armitage SAO. 2016 Maximising fitness in the face of parasites: a review of host tolerance. Zoology **119**, 281-289. (10.1016/j.zool.2016.05.011) 27373338

[91] Bernardo-Cravo AP, Schmeller DS, Chatzinotas A, Vredenburg VT, Loyau A. 2020 Environmental factors and host microbiomes shape host–pathogen dynamics. Trends Parasitol. **36**, 616-633. (10.1016/j.pt.2020.04.010) 32402837

[92] McLaren MR, Callahan BJ. 2020 Pathogen resistance may be the principal evolutionary advantage provided by the microbiome. Phil. Trans. R. Soc. B **375**, 20190592. (10.1098/rstb.2019.0592) 32772671 PMC7435163

[93] Kohl KD, Carey HV. 2016 A place for host–microbe symbiosis in the comparative physiologist's toolbox. J. Exp. Biol. **219**, 3496-3504. (10.1242/jeb.136325) 27852759

[94] Rebollar EA et al. 2016 Using "omics" and integrated multi-omics approaches to guide probiotic selection to mitigate chytridiomycosis and other emerging infectious diseases. Front. Microbiol. **7**, 68. (10.3389/fmicb.2016.00068) 26870025 PMC4735675

[95] Jin Song S, Woodhams DC, Martino C, Allaband C, Mu A, Javorschi-Miller-Montgomery S, Suchodolski JS, Knight R. 2019 Engineering the microbiome for animal health and conservation. Exp. Biol. Med. **244**, 494-504. (10.1177/1535370219830075) PMC6547002 30776908

[96] Kaganer AW, Ossiboff RJ, Keith NI, Schuler KL, Comizzoli P, Hare MP, Fleischer RC, Gratwicke B, Bunting EM. 2023 Immune priming prior to pathogen exposure sheds light on the relationship between host, microbiome and pathogen in disease. R. Soc. Open Sci. **10**, 220810. (10.1098/rsos.220810) 36756057 PMC9890126

[97] Singh RK et al. 2017 Influence of diet on the gut microbiome and implications for human health. J. Transl. Med. **15**, 73. (10.1186/s12967-017-1175-y) 28388917 PMC5385025

[98] Youngblut ND, Reischer GH, Walters W, Schuster N, Walzer C, Stalder G, Ley RE, Farnleitner AH. 2019 Host diet and evolutionary history explain different aspects of gut microbiome diversity among vertebrate clades. Nat. Commun. **10**, 2200. (10.1038/s41467-019-10191-3) 31097702 PMC6522487

[99] Dalile B, Van Oudenhove L, Vervliet B, Verbeke K. 2019 The role of short-chain fatty acids in microbiota–gut–brain communication. Nat. Rev. Gastroenterol. Hepatol. **16**, 461-478. (10.1038/s41575-019-0157-3) 31123355

[100] Warne RW, Dallas J. 2022 Microbiome mediation of animal life histories via metabolites and insulin-like signalling. Biol. Rev. **97**, 1118-1130. (10.1111/brv.12833) 35043537

[101] Hughey MC, Warne R, Dulmage A, Reeve RE, Curtis GH, Whitfield K, Schock DM, Crespi E. 2023 Diet- and salinity-induced modifications of the gut microbiota are associated with differential physiological responses to ranavirus infection in *Rana sylvatica*. Phil. Trans. R. Soc. B **378**, 20220121. (10.1098/rstb.2022.0121) 37305908 PMC10258663

[102] Martin LB et al. 2019 Extreme competence: keystone hosts of infections. Trends Ecol. Evol. **34**, 303-314. (10.1016/j.tree.2018.12.009) 30704782 PMC7114649

[103] Paull SH, Song S, McClure KM, Sackett LC, Kilpatrick AM, Johnson PTJ. 2012 From superspreaders to disease hotspots: linking transmission across hosts and space. Front. Ecol. Environ. **10**, 75-82. (10.1890/110111) 23482675 PMC3589764

[104] Longo AV, Lips KR, Zamudio KR. 2023 Evolutionary ecology of host competence after a chytrid outbreak in a naive amphibian community. Phil. Trans. R. Soc. B **378**, 20220130. (10.1098/rstb.2022.0130) 37305909 PMC10258670

[105] Brock PM, Murdock CC, Martin LB. 2014 The history of ecoimmunology and its integration with disease ecology. Integr. Comp. Biol. **54**, 353-362. (10.1093/icb/icu046) 24838746 PMC4184350

[106] Schoenle LA, Downs CJ, Martin LB. 2018 An introduction to ecoimmunology. In Advances in comparative immunology (ed. EL Cooper), pp. 901-932. Cham, Switzerland: Springer International Publishing.

[107] Downs CJ, Adelman JS, Demas GE. 2014 Mechanisms and methods in ecoimmunology: integrating within-organism and between-organism processes. Integr. Comp. Biol. **54**, 340-352. (10.1093/icb/icu082) 24944113

[108] Macknight NJ, Dimos BA, Beavers KM, Muller EM, Brandt ME, Mydlarz LD. 2022 Disease resistance in coral is mediated by distinct adaptive and plastic gene expression profiles. Sci. Adv. **8**, eabo6153. (10.1126/sciadv.abo6153) 36179017 PMC9524840

[109] Demas GE, Carlton ED. 2015 Ecoimmunology for psychoneuroimmunologists: considering context in neuroendocrine–immune–behavior interactions. Brain. Behav. Immun. **44**, 9-16. (10.1016/j.bbi.2014.09.002) 25218837 PMC4275338

[110] White TA, Perkins SE. 2012 The ecoimmunology of invasive species. Funct. Ecol. **26**, 1313-1323. (10.1111/1365-2435.12012)

[111] Rodriguez KM, Voyles J. 2020 The amphibian complement system and chytridiomycosis. J. Exp. Zool. A Ecol. Integr. Physiol. **333**, 706-719. (10.1002/jez.2419) 33052039 PMC7821119

[112] Brown GP, Hudson CM, Shine R. 2023 Do changes in body mass alter white blood cell profiles and immune function in Australian cane toads (*Rhinella marina*)? Phil. Trans. R. Soc. B **378**, 20220122. (10.1098/rstb.2022.0122) 37305913 PMC10258668

[113] Titon SCM, Junior BT, Assis VR, Cobo de Figueiredo A, Floreste FR, Lima AS, Gomes FR. 2023 Testosterone immunomodulation in free-living and captive *Rhinella icterica* male toads. Phil. Trans. R. Soc. B **378**, 20220118. (10.1098/rstb.2022.0118) 37305916 PMC10258661

[114] Carey C. 2005 How physiological methods and concepts can be useful in conservation biology. Integr. Comp. Biol. **45**, 4-11. (10.1093/icb/45.1.4) 21676738

[115] Ficetola GF. 2015 Habitat conservation research for amphibians: methodological improvements and thematic shifts. Biodivers. Conserv. **24**, 1293-1310. (10.1007/s10531-015-0869-9)

[116] Falaschi M, Melotto A, Manenti R, Ficetola GF. 2020 Invasive species and amphibian conservation. Herpetologica **76**, 216. (10.1655/0018-0831-76.2.216)

[117] Kohli AK, Lindauer AL, Brannelly LA, Ohmer MEB, Richards-Zawacki C, Rollins-Smith L, Voyles J. 2019 Disease and the drying pond: examining possible links among drought, immune function, and disease development in amphibians. Physiol. Biochem. Zool. **92**, 339-348. (10.1086/703137) 30990770

